# Gaps in detailed knowledge of human papillomavirus (HPV) and the HPV vaccine among medical students in Scotland

**DOI:** 10.1186/1471-2458-13-264

**Published:** 2013-03-22

**Authors:** Sarah M McCusker, Ishbel Macqueen, Graham Lough, Alasdair I MacDonald, Christine Campbell, Sheila V Graham

**Affiliations:** 1 MRC-University of Glasgow Centre for Virus Research, Institute of Infection Immunity and Inflammation, College of Medical, Veterinary and Life Sciences, University of Glasgow, Glasgow, Scotland, G12 8TT, UK; 2 Centre for Population Health Sciences, The University of Edinburgh, Room 309, Doorway 1, Medical Quad, Teviot Place, Edinburgh, Scotland, EH8 9AG, UK

**Keywords:** Human papillomavirus, Knowledge, Vaccine, Medical students, Public health

## Abstract

**Background:**

A vaccination programme targeted against human papillomavirus (HPV) types16 and 18 was introduced in the UK in 2008, with the aim of decreasing incidence of cervical disease. Vaccine roll out to 12–13 year old girls with a catch-up programme for girls aged up to 17 years and 364 days was accompanied by a very comprehensive public health information (PHI) campaign which described the role of HPV in the development of cervical cancer.

**Methods:**

A brief questionnaire, designed to assess acquisition of knowledge of HPV infection and its association to cervical cancer, was administered to two different cohorts of male and female 1^st^ year medical students (school leavers: 83% in age range 17–20) at a UK university. The study was timed so that the first survey in 2008 immediately followed a summer's intensive PHI campaign and very shortly after vaccine roll-out (150 students). The second survey was exactly one year later over which time there was a sustained PHI campaign (213 students).

**Results:**

We addressed three research questions: knowledge about three specific details of HPV infection that could be acquired from PHI, whether length of the PHI campaign and/or vaccination of females had any bearing on HPV knowledge, and knowledge differences between men and women regarding HPV. No female student in the 2008 cohort had completed the three-dose vaccine schedule compared to 58.4% of female students in 2009. Overall, participants’ knowledge regarding the sexually transmitted nature of HPV and its association with cervical cancer was high in both year groups. However, in both years, less than 50% of students correctly identified that HPV causes over 90% of cases of cervical cancer. Males gave fewer correct answers for these two details in 2009. In 2008 only around 50% of students recognised that the current vaccine protects against a limited subset of cervical cancer-causing HPV sub-types, although there was a significant increase in correct response among female students in the 2009 cohort compared to the 2008 cohort.

**Conclusions:**

This study highlights a lack of understanding regarding the extent of protection against cervical cancer conferred by the HPV vaccine, even among an educated population in the UK who could have a vested interest in acquiring such knowledge. The intensive PHI campaign accompanying the first year of HPV vaccination seemed to have little effect on knowledge over time. This is one of the first studies to assess detailed knowledge of HPV in both males and females. There is scope for continued improvements to PHI regarding the link between HPV infection and cervical cancer.

## Background

Anogenital human papillomavirus (HPV) infection is the most prevalent viral sexually transmitted infection (STI) in the world today [1]. Infection is extremely common, most prevalent among sexually active young adults and usually follows a benign course. Persistent infection with “high risk” (HR) HPV causes cervical disease that can lead to cervical cancer [2]. HR-HPV types, of which there are 15, cause over 99% of cervical cancers [3].

September 2008 saw the introduction of a UK nationwide HPV immunisation programme [4]. The vaccine is now offered routinely in secondary school, to girls aged 12 – 13 years. A three-year “catch up” campaign, delivered in GP surgeries and dedicated vaccination centres, has allowed older girls up to the age of 17 and 364 days access to the vaccine. Boys are not vaccinated. In the UK, between 2008 and 2012, the bivalent Cervarix vaccine [5] was used to protect against the two HR-HPV types, HPV 16 and 18, that cause around 70% of cervical cancers [1]. The vaccine was provided in three doses over six months to elicit a protective immune response in vaccinees prior to exposure to the virus [6].

Vaccine uptake in the UK has been excellent, at > 90% for 12–13 year-old girls. However, uptake rates for the catch-up programme are lower at less than 60% overall [7]. Vaccination is accompanied by targeted public health information (PHI), in the form of leaflets and/ or information sharing sessions directed by trained school nurses. Details of HPV infection and its association with cervical cancer are explained: HPV is sexually transmitted, HPV causes all cervical cancers, HPV vaccine protects against two types of HPV. Therefore, young women who have been vaccinated may be expected to have increased knowledge of HPV and its association with cervical cancer compared to the unvaccinated population. At the launch of the vaccination programme these routine information sources were supplemented by an extremely intensive media campaign as well as a specially developed Scottish website (http://www.fightcervicalcancer.org.uk/) and associated literature. The website has now been withdrawn and replaced with information on immunisation (http://www.immunisationscotland.org.uk/vaccines-and-diseases/hpv.aspx). Although these efforts were largely targeted to women, young men would also have been expected to have some exposure to the media campaign because of its intensity and range (cinema, television, radio, billboards, newspaper adverts) in 2008 [8].

Correct knowledge about HPV infection and cervical cancer could be important to inform decision making regarding uptake of the vaccine. Prior to vaccine roll out many studies revealed a lack of awareness of HPV (i.e. whether respondents had heard of the virus) and a lack of knowledge of the association between HPV and cervical cancer in the UK and a range of other countries [9-14]. Recent studies have demonstrated increased awareness of HPV following the introduction of the vaccine [15,16]. However, fewer studies have investigated acquisition of detailed knowledge of HPV infection. Those that have, indicate low levels of more detailed knowledge of HPV infection and links to cervical cancer [17-20]. Such knowledge appears to be greatest in those with higher education levels [19].

This study was conducted at the University of Glasgow, Scotland. Prospective medical students at this university are admitted to study on the basis of interview where depth of knowledge of current topics in medicine is assessed. The HPV vaccine was one of the main topics for 2008 and 2009 so new medical students at the university could be expected to acquire some knowledge of HPV. HPV knowledge was assessed in two groups of first year medical students, mean age range 17–20 years of age. The timings of the surveys were specifically chosen. The first survey was carried out in November 2008 immediately following the roll-out of the HPV vaccine and a summer’s intensive media campaign that aimed to inform the public about HPV and the HPV vaccine. The second cohort was surveyed one year later when many more females had received the vaccine and associated PHI. The multimedia PHI campaign was sustained over this one year period. Moreover, media interest in the death from cervical cancer of the celebrity Jade Goody [21,22], and increased discussion of HPV in the media, at the start of 2009 coincided with the timing of interviews for medical school of the 2009 student cohort.

We sought to answer three research questions. The first question we addressed was knowledge about three specific details of HPV infection that could be acquired from reading of PHI leaflets accompanying the HPV vaccination programme and from PHI in an intensive media campaign between 2008 and 2009. The second research question was whether vaccination of females in the group and a year’s PHI campaign had any bearing on HPV knowledge. In contract to policy in the rest of the UK where screening starts at age 25, from age 20, Scottish (and Welsh) women are offered three-yearly cervical screening for the detection of HPV-associated cervical disease. 73.7% of eligible women were screened in the past three and a half years (as of March 31^st^, 2010), which is a rise in uptake of around 4.5 percent in comparison to previous years [23]. Arguments for and against screening before the age of 25 can be found at http://www.cks.nhs.uk/cervical_screening/management/scenario_when_to_offer_cervical_screening/. The final research question was to find out if men and women acquired similar levels of knowledge about HPV from the same or different sources. This study is among the first to compare knowledge of details of HPV of an educated mixed gender cohort at a time of an extremely intensive media campaign then exactly one year later in a similar cohort where most of the females had received at least one dose of the HPV vaccine and following a sustained PHI campaign.

## Methods

### Study design

A questionnaire survey was administered to first year medical students at a UK university (University of Glasgow). Medical students were chosen as a "best case scenario" of HPV knowledge acquisition among young adults because they are an educated population and may have a greater awareness of current medical issues: entry into medical school at Glasgow is by interview where prospective students are questioned on knowledge of current topics in human health. Moreover, as health professionals of the future, they may have high motivation to pay attention to and acquire current PHI. Student knowledge and opinion was assessed in 2008 in that year’s intake of medical students i.e. immediately following the implementation of HPV vaccine delivery in the UK, and then one year later in the 2009 intake of students. The same questionnaire was administered in both years.

### Survey questionnaire

An 8-item closed-ended questionnaire, with, for females, a 3-item data gathering section to determine stage of vaccination, intention to attend smear testing and smear testing status, was developed from a literature review and in consultation with a vaccine specialist at Health Protection Scotland and NHS public health information specialists. No studies examining detailed knowledge of HPV in the UK were reported in the literature at the time of this study so three questions relating to HPV were designed to address specific points regarding HPV knowledge discussed in leaflets delivered to vaccinees and in other PHI. The draft questionnaire was piloted on students in the final year honours virology classes of both years. The questionnaire was then reviewed by members with relevant expertise of the University of Glasgow Ethics Committee and modified according to feedback received. The survey was administered at the same time of year, in November, first in 2008 and then in 2009. This timing was chosen because it preceded taught modules on cancer, including cervical cancer. The questionnaire was completed in a ten minute period at the end of a teaching session in a lecture theatre. One of the researchers introduced the study and explained the ethical permissions and voluntary nature of participation. Students made aware of how anonymity would be ensured. Although signed informed consent was given, the consent forms were collected separately to the questionnaires so that no questionnaire answers could be traced back to an individual. Ethical approval was obtained from the University of Glasgow’s Faculty of Biomedical and Life Sciences Ethics Committee.

In 2008 the timing of the survey was chosen to be immediately following first roll out of the vaccine (September 2008) after a summer's intensive public health campaign. This included television, radio, internet, media, cinema and billboard adverts and age-directed leaflets available in public places, community and health centres and GP surgeries. Few females in the population were vaccinated in 2008. In contrast, in 2009, many of the female students had been offered the vaccine, delivered in association with appropriate PHI. Moreover, there was a sustained high level PHI campaign throughout 2009. In particular, during this period there was extensive media coverage of the illness and death due to cervical cancer of the celebrity Jade Goody [21,22].

### The study group

In 2008 all 150 students (100%) and 213 out of 217 students in 2009 (98.2%) who attended the taught sessions at which the questionnaires were given out responded to the questionnaire. In 2008 the survey population consisted of 100 female and 50 male participants while in 2009 the survey population contained 114 female and 99 male students. In both survey populations the majority of students were between seventeen and twenty years of age (84% in 2008; 83% in 2009) (Table 1). In 2008, 90% and in 2009, 87% of students were from the UK. In 2008, 91% of the female students had not been vaccinated and no student had received all three vaccine doses. In contrast, in 2009 58.4% of female students had received three vaccine doses (Table 1). All students who filled in the questionnaire did so completely except for up to two non-responders to questions regarding uptake of smear testing.

**Table 1:** Age ranges of the study populations (2008: n=150, 2009: n=213) and vaccination status of female respondents

**Year**	**2008**			**2009**		
	**Female**		**Male**	**Female**		**Male**
Age group	(n)		(n)	(n)		(n)
17-20	83		43	97		80
21-25	10		3	11		14
>25	7		4	6		5
Total	100		50	114		99
Vaccination status	% (n)		N/A	% (n)		N/A
Not vaccinated	91	(91)		38.3	(43)	
Had 1^st^ injection	2	(2)		0.9	(1)	
Had 1^st^ and 2^nd^ injections	4	(4)		0	(0)	
Had all 3 injections	0	(0)		58.4	(66)	
Do not wish vaccination	3	(3)		2.4	(3)	

N/A=not applicable.

### Data collation and analysis

Data were input into Microsoft Excel 11.5. Exact binomial, Randomisation and Chi-Squared tests were used to compare between group data. Differences were considered significant if p< 0.05. Analyses focused on questions pertaining to knowledge of HPV and the HPV vaccine.

## Results

### Knowledge of details of HPV and its association with cervical cancer

In 2008, 97.3% of the class marked as true the statement "HPV is sexually transmitted between males and females" (Table 2). Only four students did not consider that HPV is sexually transmitted between males and females. However, in 2009, 10% of students did not consider that HPV was sexually transmitted indicating a decrease in knowledge acquisition, especially in the male population, in the second group of students. The medical students were asked to estimate what percentage of cervical cancers is due to HPV. Less than half of all males and females in both survey populations answered correctly. There was some difference in the proportion of correct answers given by males in 2009 compared to 2008 (Table 2). When asked the question "There are at least 15 different types of HPV that can cause cervical cancer. How many of these types do you think the vaccine protects against?" the majority of female students in 2008 and 2009 answered correctly that the vaccine protects against less that 5 HPV types. There was a statistically significant positive difference in the response of females to this question over time (73% answered correctly in 2009 compared to 50% of females in 2008) (Table 2). Male responses in both years were very similar to the 2008 female responses and showed no statistical difference comparing responses in 2008 with 2009.

**Table 2:** Frequency of responses to questions in the questionnaire in 2008 and 2009: Students' knowledge of HPV and cervical cancer

	**2008**		**2009**	
	**Female**	**Male**	**Female**	**Male**
	**% (n)**	**% (n)**	**% (n)**	**% (n)**
Q1: HPV is sexually transmitted between males and females:				
True	96 (96)	100 (50)	92 (105)	86 (86)
False	4 (4)	0	8 (9)	13 (13)*
Q2: What percentage of cervical cancers do you think HPV is responsible for?				
<20%	6 (6)	4 (2)	3 (3)	10 (10)*
20-80%	50 (50)	50 (25)	58 (66)	54 (54)
80-100%	44 (44)	46 (23)	39 (45)	35 (35)*
Q3: There are at least 15 types of HPV that can cause cancer. How many of these types do you think the HPV vaccine protects against?				
0-5 HPVs	50 (50)	52 (26)	73 (83)*	51 (50)
5-10 HPVs	36 (36)	30 (15)	24 (27)*	39 (39)
10-15 HPVs	14 (14)	18 (9)	3 (4)*	10 (10)

*= p-value <0.05: significant difference between 2008 and 2009 between the same gender.

### Assessing potential impact of the vaccine on uptake of cervical smear testing

In 2008, 94% and in 2009, 87.3% of the students assessed the cervical screening programme as “very important”. In Scotland, women are invited for their first smear test at age 20. Some of the female students in the survey populations had already attended for testing: in 2008, 16.0% (n=16) of students (97% of those eligible for screening (i.e. ≥20 years of age)) had been for a smear test while in 2009, 13.3% (n=15) (68% of those eligible for screening) had been tested. In both years the majority of women answered positively when asked if they would go for a smear test when invited. Importantly, in 2009, 94% of females who had been fully vaccinated said they would attend for a smear test when invited.

### Exposure to public health information on the HPV vaccine

The impact of the HPV vaccine campaign was also examined (Table 3). Most students, male and female, in both years claimed to have been exposed to PHI on the HPV vaccine, reporting television advertising and word of mouth as the main source in the 2008 survey. The 2009 survey included more options due to an increased range of PHI resources available since the start of the vaccination programme and most students reported having seen these. However, in this second year, a much lower percentage of male students reported receiving information from television adverts but a greater percentage reported receiving information by word of mouth. More males in this year were unable to rate PHI on the HPV vaccine.

**Table 3:** Participants’ rating of public health information (PHI) on the HPV vaccine

**Year**	**2008**		**2009**	
	**Female**	**Male**	**Female**	**Male**
	**% (n)**	**% (n)**	**% (n)**	**% (n)**
Q1. How would you rate the PHI on the HPV vaccine?				
Poor	10 (10)	20 (10)	7 (6)	9 (9)
Not bad	64 (64)	60 (30)	46 (54)	35 (36)
Very good	25 (25)	18 (9)	43 (49)	33 (33)
Don’t know	1 (1)	2 (1)	4 (5)	22 (22)
Q2. Where have you heard/seen information on the current HPV vaccination programme? Tick all that apply.				
TV advertisement	72 (72)	66 (33)	79 (90)	23 (23)
Radio advertisement	24 (24)	18 (9)	31 (35)	30 (30)
Information leaflet	34 (34)	28 (14)	55 (63)	47 (48)
Poster in a pharmacy			45 (51)	50 (50)
Internet			42 (48)	41 (41)
School/College/University	42 (42)	62 (31)	75 (85)	68 (68)
GP/Doctor	26 (26)	8 (4)	18 (21)	2 (2)
Word of mouth	71 (71)	78 (39)	63 (72)	77 (77)
I have not seen/heard anything	1 (1)	2 (1)	3 (3)	3 (3)
Other*			3 (4)	5 (5)

* reported as newspapers, clinical journals.

## Discussion

### Summary

Many studies in different countries in a range of socio-economic groups have demonstrated an overall lack of awareness of HPV and its link with cervical cancer [9-11,16,24,25]. This study represents one of the first reports of the knowledge of educated young people in the UK of details about HPV infection, the HPV vaccine and cervical cancer, and importantly, how knowledge changed within the first year of introduction of the vaccination programme. The surveys were carefully timed to question the students when they would have been maximally exposed to PHI and media information on HPV and the HPV vaccine. Moreover, the students may have had more motivation to pay attention to details in the PHI than most other young people of this age group because their successful entry to medical school could depend on their displaying clear knowledge and understanding of facts on HPV at interview. There are few studies comparing detailed knowledge of HPV in males and females. The young male population could have a role in encouraging their female peers to be vaccinated so both genders were questioned in each year’s cohort. The study included unvaccinated women and women who were in the age group between being offered the vaccine and being called for cervical screening. The vast majority (99%) of the students, male and female, questioned in each year indicated that they had seen or heard information on the HPV vaccine indicating some efficacy of PHI. The questionnaire assumed that the students would have some knowledge of HPV and cervical cancer and indeed it was clear that the level of knowledge of this educated population was probably greater than that of the general population [10] as recently suggested [26].

### Knowledge about specific details of HPV infection

The UK PHI leaflets accompanying HPV vaccination note the sexually transmitted nature of HPV. Surprisingly, although most students seemed to appreciate this fact, in agreement with another study [27], the 2009 survey showed a small decrease in the percentage of students who understood that HPV was sexually transmitted. This was despite a significant step-up in the PHI campaign surrounding HPV and its link with cervical cancer from the summer of 2008 through to autumn 2009 and the media coverage early in 2009 of the death from cervical cancer of the young celebrity Jade Goody [21,22]. However, the level of knowledge of the sexually-transmitted nature of HPV in our cohorts was much higher than that in another study of a similar age group of school students in Germany where less than 50% understood that HPV was a sexually transmitted infection [28]. It is becoming increasingly clear from similar surveys in a number of countries that even among educated young people there is a lack of understanding of the sexually transmitted nature of HPV infection [18,19,29-31].

PHI leaflets clearly state that the vaccine protects against 70% of cases of cervical cancer. However, it is not made clear that the remaining 30% of cases are caused by *other* HR-HPV types. Consequently, anecdotal evidence suggests that the public may believe it is possible to develop cervical cancer without being infected with HPV [16,32,33]. Students were asked to guess what percentage of cervical cancers was caused by HPV. Only around half of men and women in 2008 understood that HPV caused between 80 and 100% of cervical cancers. There was a decrease in understanding this fact between the 2008 and 2009 surveys and a greater percentage of men responded incorrectly in 2009.

### Relationship between vaccination and HPV knowledge

The vaccine PHI intended to inform the public that the vaccine protected against two types of HPV. However, in 2008 around half the survey population did not appear to appreciate this fact. Male understanding did not increase by 2009 but females gave a larger number of correct responses in that year. This could be due to the increased percentage of vaccinated female students in 2009 that correlated with an increased number who claimed to have seen or heard HPV information in formal education, as expected due to the PHI delivered to vaccinees. However, as noted above, a lower percentage of the 2009 cohort of women realised that HPV caused most cervical cancers. Therefore our evidence is equivocal that PHI delivered with the vaccine improved HPV knowledge.

### Acquiring knowledge: gender differences in HPV knowledge

Although there was some evidence of gender differences in HPV knowledge, especially in the 2009 cohort, gender did not have a significant difference on the sources of information on HPV. However, a much higher percentage of male students reported receiving information from word of mouth and more males in 2009 compared to 2008 were unable to rate PHI on the HPV vaccine. Interestingly, although the males in the study displayed knowledge of HPV association with cervical cancer, the majority answered ‘no’ to a question included in the survey “Do you think males should be offered the vaccine?” This could indicate perception of HPV vaccination as a female issue.

### Limitations of the study

The study has some limitations. Prior to administering the questionnaire it is possible that some students had begun to prepare for the oncology portion of their course by reading recommended materials and had thus acquired a greater appreciation of the subject prior to questioning. Some homogenisation of answers could have occurred through students comparing answers in class. The restricted time period (ten minutes) for filling in the questionnaire was designed to avoid this. In itself this may have introduced another limitation because the short time period may have led the students to provide rapid, poorly considered responses.

## Conclusions

Public knowledge of HPV, cervical cancer and vaccination is central to ensuring good vaccination coverage in the female population and success of future HPV testing strategies that will reduce the burden of disease. This survey indicates that PHI has been somewhat successful in delivering key elements of HPV knowledge for both genders in the young educated population studied, but that there are still important knowledge gaps especially in males. Continued efforts in pressing home the main messages regarding HPV, the HPV vaccine, and cervical screening are required.

### Ethical approval

University of Glasgow Faculty of Biomedical Sciences Ethics Committee project approval number FBLS 0825 17/11/2008 and FBLS 0930 01/10/2009.

## Abbreviations

HPV: Human papillomavirus; PHI: Public health information; STI: Sexually transmitted infection

## Competing interests

We declare no conflict of interest.

## Authors’ contributions

SMcC compared the data analytically from both cohorts and wrote the first draft of the paper. GL conducted the survey and analysed data in 2008. IM conducted the survey and analysed data in 2009. AMcD carried out the statistical analyses. CC advised on analysis and co-wrote the paper. SG supervised the study and co-wrote the paper. All authors read and approved the final manuscript.

## Pre-publication history

The pre-publication history for this paper can be accessed here:

http://www.biomedcentral.com/1471-2458/13/264/prepub
